# *In Vitro* Blood-Brain Barrier Models Using Brain Capillary Endothelial Cells Isolated from Neonatal and Adult Rats Retain Age-Related Barrier Properties

**DOI:** 10.1371/journal.pone.0055166

**Published:** 2013-01-31

**Authors:** Fuyuko Takata, Shinya Dohgu, Atsushi Yamauchi, Junichi Matsumoto, Takashi Machida, Kayoko Fujishita, Keisuke Shibata, Youichi Shinozaki, Kaoru Sato, Yasufumi Kataoka, Schuichi Koizumi

**Affiliations:** 1 Department of Pharmaceutical Care and Health Sciences, Faculty of Pharmaceutical Sciences, Fukuoka University, Fukuoka, Japan; 2 BBB Laboratory, PharmaCo-Cell Co., Ltd., Nagasaki, Japan; 3 Department of Neuropharmacology, Interdisciplinary Graduate School of Medicine and Engineering, University of Yamanashi, Yamanashi, Japan; 4 Japan Science and Technology Agency, Core Research for Evolutional Science and Technology, Tokyo, Japan; 5 Division of Pharmacology, National Institute of Health Sciences, Tokyo, Japan; Institute of Neurology (Edinger-Institute), Germany

## Abstract

The blood–brain barrier (BBB) restricts the entry of circulating drugs and xenobiotics into the brain, and thus its permeability to substances is a critical factor that determines their central effects. The infant brain is vulnerable to neurotoxic substances partly due to the immature BBB. The employment of *in vitro* BBB models to evaluate permeability of compounds provides higher throughput than that of *in vivo* animal experiments. However, existing *in vitro* BBB models have not been able to simulate the intrinsic neonatal BBB. To establish a neonatal BBB model that mimics age-related BBB properties, the neonatal and adult *in vitro* BBB models were constructed with brain endothelial cells isolated from 2- and 8-week-old rats, respectively. To evaluate BBB functions, transendothelial electrical resistance, permeability of sodium fluorescein and Evans blue-albumin, and transport of rhodamine123 were measured. Radiolabelled drugs were used for BBB permeability studies in the neonatal and adult BBB models (*in vitro*) and in age-matched rats (*in vivo*). The neonatal BBB model showed lower barrier and p-glycoprotein (P-gp) functions than the adult BBB model; these were well associated with lower expressions of the barrier-related proteins and P-gp, and a different distribution pattern of immunostained barrier-related proteins. Verapamil (a P-gp inhibitor) significantly increased the influx of rhodamine 123, supporting functional P-gp expression in the neonatal BBB model. Valproic acid, but not nicotine, showed higher BBB permeability in the neonatal BBB model, which was well in accordance with the *in vivo* BBB property. We established a neonatal BBB model *in vitro*. This could allow us to assess the age-dependent BBB permeability of drugs.

## Introduction

The developing brain is known to be vulnerable to neurotoxic substances including therapeutic drugs. During pregnancy, a large number of women take one or more drugs [1]. Especially, psychotropic drugs taken by an expectant mother readily cross the placenta to reach the fetus. As well as prenatal exposure to potentially neurotoxic substances, postnatal exposure can affect brain development. The blood-brain barrier (BBB) develops with age to acquire the barrier function and protect the brain parenchyma from blood-borne neurotoxic substances. The BBB is comprised of brain microvessel endothelial cells (BMECs) and perivascular cells (pericytes and astrocytes). This barrier functions as an interface between the brain parenchyma and circulating blood. Brain capillaries form a layer that prevents xenobiotics from entering into the brain due to tight junctions between BMECs and efflux transporters on BMECs. Despite the presence of brain capillaries, the vulnerability to neurotoxic substances has been known to differ between adult and developing brains [2], [3], [4]. This phenomenon is attributed to degree of maturity of the BBB. For example, oseltamivir, an antiviral drug, shows neuropsychiatric adverse events in children, provably due to direct effects on the CNS [5]. A possible explanation for these adverse effects in children is that BBB permeability to oseltamivir changes with development. There are reports showing that the BBB in newborn animals allows compounds with low BBB permeability, in the adult brain, to penetrate into the brain parenchyma [6], [7]. This suggests that ontogenic differences in endothelial barrier properties at the BBB may account for different responses to drugs in the CNS between infant and adult humans. Thus determining the permeability of individual drugs through the infant BBB is required to predict and/or evaluate the potential neurotoxicity.

*In vitro* BBB models have contributed to our knowledge of the physiology, pathology and pharmacology of the BBB [8]. The employment of *in vitro* BBB models to evaluate the direct effect and permeability of many compounds provides higher throughput than that of *in vivo* animal experiments. Although monolayer cultures of brain microvascular endothelial cells isolated from various species (human, bovine, porcine, rat, and mouse) with a porous membrane insert are widely used as *in vitro* BBB models, these monolayer models lack critical features including cell-to-cell interaction with perivascular cells, i.e., pericytes and astrocytes. These cells potentiate the endothelial barrier properties by inducing the expression of tight junction proteins and various efflux transporters on BMECs [9], [10], [11], [12]. It has recently been reported that a new *in vitro* BBB model using rat BMECs, pericytes, and astrocytes shows a good correlation between the *in vitro* endothelial permeability coefficient (Pe) value obtained from this rat triple co-culture model and the *in vivo* BBB permeability data with 19 drugs [13]. Therefore, reproducing the cellular interaction among BMECs, pericytes and astrocytes improves accuracy when predicting the *in vivo* BBB permeability from *in vitro* BBB models.

A previous *in vivo* study showed that barrier functions in the developing brain are less effective than those in the adult brain [14], [15], [16]. To predict the permeability of xenobiotics including therapeutic drug candidates through the BBB in the infant using *in vitro* BBB models, age-related changes in the BBB have to be considered. Animals of various ages were used to reconstitute the BBB *in vitro*. In a rat BBB model, 2- [17], 3- [11], [13], [18], [19], [20], and 8- to 12- [21] week-old rats were used to isolate BMECs. However, it is not known whether age-related changes in the BBB functions *in vivo* are reproducible in the primary culture of BMECs isolated from animals of various ages.

In the present study, we first tested whether *in vitro* BBB models using BMECs isolated from neonatal (2-week-old) and adult (8-week-old) rats show age-related differences in the BBB properties and permeability of drugs. Second, to test whether this neonatal BBB model *in vitro* would be useful for prediction of the neonatal BBB permeability *in vivo*, we compared the *in vitro* drug permeability with that obtained from the *in vivo* experiments using rats with each corresponding age.

## Materials and Methods

### Reagents

[^3^H] valproic acid (20 Ci/mmol), [^3^H] nicotine (85 Ci/mmol) and [^14^C] inulin (2 mCi/g) were purchased from American Radiolabeled Chemicals, Inc. (St. Louis, MO, USA). Valproic acid was purchased from Sigma (St. Louis, MO, USA). Dulbecco’s modified Eagle’s medium (DMEM) and DMEM/Ham’s nutrient mixture F-12 medium (DMEM/F12) were purchased from Wako (Osaka, Japan) and Sigma, respectively. Fetal bovine serum (FBS) and plasma-derived serum (PDS) were purchased from Biowest (Nuaillé, France) and Animal Technologies Inc. (Tyler, TX, USA), respectively.

### Cell Cultures

Wistar rats at 2 and 8 weeks old were obtained from CREA Japan, Inc. (Tokyo, Japan). The rats had free access to food and water and were maintained on a 12-h dark/light cycle in a room with controlled temperature (24±1°C) and humidity (55±5%). All procedures involving experimental animals adhered to the law (No. 105) and notification (No.6) of the Japanese Government, and were approved by the Laboratory Animal Care and Use Committee of Fukuoka University.

Primary cultures of neonatal (2 weeks old) and adult (8 weeks old) rat brain capillary endothelial cells (RBECs) were prepared from two and eight weeks old Wistar rats respectively, as previously described [9], [11], [20], [22]. The meninges were carefully removed from the forebrains, and the gray matter was minced in DMEM cooled at 2°C, and digested with collagenase type 2 (1 mg/mL, Worthington, Lakewood, NJ, USA) for 1.5 h at 37°C. The pellet was separated by centrifugation in 20% bovine serum albumin (BSA)-DMEM (1000×g, 20 min). The microvessels obtained in the pellet were further digested with collagenase/dispase (1 mg/mL, Roche, Mannheim, Germany) for 1 h at 37°C. Microvessel clusters containing pericytes and endothelial cells were separated on a 33% continuous Percoll (GE Healthcare, Buckinghamshire, UK) gradient, collected and washed twice with DMEM before plating on collagen type IV-fibronectin (both 0.1 mg/mL) coated dishes. RBEC cultures were maintained in RBEC medium Ι [DMEM/F12 supplemented with 10% PDS, basic fibroblast growth factor (1.5 ng/mL, Roche), heparin (100 µg/mL, Sigma), insulin (5 µg/mL), transferrin (5 µg/mL), sodium selenite (5 ng/mL; insulin-transferrin-sodium selenite media supplement, Sigma) and gentamicin (50 µg/mL)] containing puromycin (4 µg/mL, Sigma) at 37°C in a humidified atmosphere of 5% CO_2_/95% air, for two days. To remove the puromycin, cells were washed three times with fresh RBEC medium Ι and incubated with this medium on the third day. On the fifth day, RBECs typically reached 80–90% confluency.

Primary pericyte cultures were prepared from the microvessels of 3-week-old Wistar rats according to the above-mentioned method. Microvessel clusters containing pericytes were plated on non-coated dishes (No. 353003; BD Biosciences, Franklin Lakes, NJ, USA). Brain pericyte cultures were maintained in DMEM supplemented with 20% FBS and 50 µg/mL gentamicin (Biowest). They were grown in a humidified atmosphere of 5% CO_2_/95% air at 37°C. After seven days in culture, pericytes at 80–90% confluency were used for the experiments.

Primary astrocyte cultures were prepared from the cerebral cortex of 1- to 3-day-old Wistar rats according to the method of McCarthy and de Vellis [23]with a slight modification. Briefly, after removing the meninges and blood vessels, the forebrains were minced and gently dissociated by repeated pipetting in DMEM containing 10% FBS, 100 units/mL penicillin (Nacalai Tesque, Kyoto, Japan) and 100 µg/mL streptomycin (Nacalai Tesque), and filtered through a 70-µm cell strainer. Cells were collected by centrifugation (800×g, 6 min), resuspended in 10% FBS DMEM and cultured in 75-cm^2^ flasks (BD Biosciences) in a humidified atmosphere of 5% CO_2_/95% air at 37°C. Cells were fed every 2–3 days by changing the medium. After 10–14 days in culture, floating cells and weakly attached cells of the mixed primary cultured cell layer were removed by vigorous shaking of the flask. Then, astrocytes at the bottom of the culture flask were trypsinized and seeded into new culture flasks. The primary cultured astrocytes were maintained in 10% FBS/DMEM. They were grown in a humidified atmosphere of 5% CO_2_/95% air at 37°C. Cells at the second or third passage were used for the experiments.

### Construction of *in vitro* BBB Models

Our *in vitro* BBB models were constructed according to the method of Nakagawa et al. [11], [13]. The day when the endothelial cells were plated and models were established was defined as day zero *in vitro* (day 0). To construct the *in vitro* models, pericytes (1.5×10^4^ cells/cm^2^) were seeded on the bottom side of the collagen-coated polyester membrane of Transwell inserts (Corning Life Sciences, MA, USA). The cells were allowed to adhere firmly overnight, then RBECs prepared from 2-week-old rats and 8-week-old rats (1.5×10^5^ cells/cm^2^) were seeded on the inside of the inserts placed in the well of the 24-well culture plates containing astrocytes (3×10^4^ cells/cm^2^). From day 1, BBB models were maintained in RBEC medium I except puromycin and with 500 nM hydrocortisone (RBEC medium II) [24]. Under these conditions, the *in vitro* BBB models were established within 3 days after setting of the cells. Two types of BBB models using neonatal and adult RBECs were constructed. As negative controls for barrier integrity studies, pericytes, which do not form barriers, were cultured on the insert.

### *In vitro* Evaluation of the Barrier Function

TEER, reflecting the flux of ions through cell layers in culture conditions, was measured by an Epithelial-volt-ohm meter and Endohm-6 chamber electrodes (World Precision Instruments, USA). The TEER of pericyte-layered filters was subtracted from the measured TEER values of the models, shown as Ω×cm^2^. The flux of sodium fluorescein (Na–F) and Evan’s blue-albumin (EBA) across the endothelial cell layers of the *in vitro* BBB models was determined as previously described [25]. Cell culture inserts were transferred to 24-well plates containing 0.6 ml permeability assay buffer (141 mM NaCl, 2.8 mM CaCl_2_, 1 mM MgSO_4_, 4 mM KCl, 1 mM NaH_2_PO_4_, 10 mM glucose and 10 mM Hepes, pH 7.4) in the lower or abluminal compartments. In the inserts (luminal compartment), culture medium was replaced by 0.1 ml buffer containing 100 µg/ml Na–F (MW: 376 Da) and 4% bovine serum albumin (Sigma) mixed with 0.67 mg/mL Evan’s blue dye (Sigma) (EBA; MW: 67,000 Da). Samples (400 µL) were removed from each side at 15, 30, 45, 60, 120 and 180 min. To ensure mixing of the layers, we stirred the assay buffer in the receiver chamber, into which test compounds permeate, with a pipette before removing the buffer and immediately replacing with fresh permeability assay buffer. The concentrations of Na-F were determined with a CytoFluor Series 4000 fluorescence multiwall plate reader (PerSeptive Biosystems) using a fluorescein filter pair (Ex(λ) 485±10 nm; Em(λ) 530±10 nm). The EBA concentration in the abluminal chamber was measured by determining the absorbance of samples at 630 nm with an amicroplate reader (Opsys MR, DYNEX Technologies, Chantilly, VA,USA). Flux across the pericyte culture inserts was also measured. The transendothelial permeability coefficient P_trans_ was calculated as described in Analysis of *in vitro* permeability data.

### *In vitro* Functional Assay for P-glycoprotein (P-gp)

The activity of P-gp was determined by measuring the influx (luminal-to-abluminal direction) and efflux (abluminal-to-luminal direction) of rhodamine 123, a substrate of P-gp, across the endothelial cell layers of the *in vitro* BBB models [26]. To initiate the transport experiments, the medium was removed and 5 µM rhodamine 123 in the permeability assay buffer was put on the inside (luminal) or outside (abluminal) of the inserts containing the cell layers in the well. The permeability assay buffer was loaded in the site opposite side with rhodamine 123. The flux of 5 µM rhodamine 123 in the permeability assay buffer was measured at 37°C in the luminal-to-abluminal and in the opposite abluminal-to-luminal directions. The samples (luminal-to-abluminal, 400 µL; abluminal-to-luminal, 66 µL) were removed from each side at 15, 30, 45, 60, 120 min and immediately replaced with fresh permeability assay buffer. To test if verapamil (a potential P-gp inhibitor) influences the influx of rhodamine 123, verapamil (20 µM) was added to the permeability assay buffer. The Rhodamine 123 content in the samples was determined by a CytoFluor Series 4000 fluorescence multiwall plate reader (PerSeptive Biosystems) using a fluorescein filter pair (Ex(λ) 485±10 nm; Em(λ) 530±10 nm). Flux across the pericyte culture inserts was also measured. The transendothelial permeability coefficient P_trans_ was calculated as described in Analysis of *in vitro* permeability data.

### *In vitro* Drug Transport Experiments

To measure the flux of valproic acid, nicotine and inulin across the endothelial cell layers of the *in vitro* BBB models, following the measurement of TEER the inserts were transferred to 24-well plates containing 0.6 ml permeability assay buffer in the lower compartments. In the luminal chambers culture medium was replaced by 0.1 ml permeability assay buffer containing the test compounds at 2 µCi/mL concentration. Samples (400 µL) were removed from each side at 10, 20, 30, 40 min and immediately replaced with fresh permeability assay buffer. The samples were prepared for scintillation counting by the addition of 10 mL of a liquid scintillation cocktail (Pico-Fluor 40; PerkinElmer, Waltham, MA, USA). The radioactivity in the samples was measured using a liquid scintillation counter (Packard model 2250CA). Flux across the pericyte culture inserts was also measured. PS_trans_ was calculated for each drug as described in analysis of *in vitro* permeability data. These calculated PS_trans_ values were corrected by the protein levels of RBECs.

### Analysis of *in vitro* Permeability Data

The permeability coefficient and the permeability clearance were calculated according to the method described by Dehouck et al. [27]. The transferred volume (µL) of tracer diffusing from the luminal to abluminal chamber or from the abluminal to luminal chamber was calculated from the initial concentration of tracer in the side of the chamber loaded with tracer and the final concentration in the opposite side of the chamber: transferred volume (µl) = [C]_A_ × *V*_A_/[C]_L_, where [C]_L_ is the initial tracer concentration in the side of chamber loaded with tracer, [C]_A_ is the tracer concentration in the opposite side of the chamber loaded with tracer and *V*_A_ is the volume of the opposite side of chamber loaded with tracer. During the experiment, the transferred volume increased linearly with time. The average volume cleared was plotted versus time, and the slope was estimated by linear regression analysis. The slope of the transferred volume curves for the *in vitro* BBB models was denoted by *PS*_app_ where *PS* is the permeability-surface area product (in µL/min). The slope of the transferred volume curve with the control membrane was denoted by *PS*_membrane_. The control membrane was the rat pericyte-layered membrane. The real *PS* value (the permeability clearance) for the *in vitro* BBB models (*PS*_trans_) was calculated from 1/*PS*_app_ = 1/*PS*_membrane_ +1/*PS*_trans_. The *PS*_trans_ values were divided by the surface area of the Transwell inserts to generate the permeability coefficient (*P*_trans_, in cm/min).

### *In vivo* Studies of Drug Permeability in Rats

The *in vivo* transport experiments were carried out by the in situ transcardiac brain perfusion technique using the methods of Banks et. al. [28]. The Wistar rats at 2 and 8 weeks old were anesthetized i.p. with 40% urethane (sigma), and, for each rat, the heart was exposed, both jugulars were severed, and the descending aorta was ligated. The perfusate, containing [^3^H] valproic acid or [^3^H] nicotine with [^14^C] inulin, was infused into the left ventricle of the heart at a rate of 2 ml/min for 0.5, 1.0, 1.5 and 2.0 min. This rate of perfusion quickly fills the vascular space in the brain without disrupting the BBB [29]. After perfusion, the brain was removed and weighed. Brains were mixed with 1 mL of tissue solubilizer (Solvable™; PerkinElmer) and incubated at 60°C for 24 h. Samples were prepared for scintillation counting by the addition of 0.2 mL of H_2_O_2_ and 10 mL of a liquid scintillation cocktail (Pico-Fluor 40). The radioactivity in the samples was measured using a liquid scintillation counter (Packard model 2250CA). Brain/perfusate ratios were calculated by dividing the radioactivity in a gram of brain by the radioactivity in a microliter of perfusate. [^3^H] Valproic acid, [^3^H] nicotine and [^14^C] inulin were diluted in prewarmed permeability assay buffer at the amounts of 0.2, 0.2 and 0.1 µCi/mL, respectively. The perfusate was freshly prepared each day.

### *In vivo* Multiple Time Regression Analysis

This method [30], [31] was used to calculate the blood-to-brain unidirectional influx rate (*K*_in_) of radiolabeled compounds into the brain. The brain/perfusate ratios (µL/g) for whole brain were calculated by the following formula:

(Brain/Perfusate ratio) = (cpm/g of brain)/(cpm/µl of perfusate).

The brain/perfusate ratios were plotted against the perfusion time. *K*_in_ was measured as the slope for the linear portion of the relation between the brain/perfusate ratios and the perfusion times (*t*). The *y*-intercept of the line represents the initial distribution volume (*V*_i_) in the brain at *t* = 0. The equation describing the linear portion of the relation between the brain/perfusate ratios and the perfusion time is the following equation:
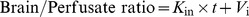



### Western Blot Analysis

Cells were scraped and lysed in lysis buffer (10 mM Tris-HCl, pH 6.8, 100 mM NaCl, 1 mM EDTA, 10% glycerol, 1% Triton X-100, 0.1% SDS, 0.5% sodium deoxycholate, 2 mM Na_3_VO_4_, 50 mM NaF, 20 mM sodium pyrophosphate decahydrate and 50 µg/mL phenylmethylsulfonyl fluoride) containing 1% phosphatase inhibitor cocktail 1 (Sigma), 1% phosphatase inhibitor cocktail 2 (Sigma) and 1% protease inhibitor cocktail (Sigma). The total protein concentration in the cell lysates was determined using a BCA Protein assay kit (Pierce, Rockford, IL, USA). Equivalent amounts of protein from each sample were electrophoretically separated on 5–20% SDS-polyacrylamide gels (Bio Craft Co., Ltd., Tokyo, Japan), and then transferred to polyvinylidene difluoride membranes (Bio-Rad Laboratories, Hercules, CA, USA). Membranes were blocked with Blocking One (Nacalai Tesque). Claudin-5, ZO-1, occludin, P-gp and β-actin were detected using antibodies against Claudin-5 (1∶1000; Invitrogen), ZO-1 (1∶250; Invitrogen), occludin (1∶100; BD transduction Laboratory, Franklin Lakes, NJ, USA), P-gp (C219; 5 µg/mL; Calbiochem, La Jolla, CA, USA), and β-actin (1∶1000; Abcam, Cambridge, UK). After washing, the membranes were incubated with an appropriate horseradish peroxidase-conjugated secondary antibody. The immunoreactive bands were visualized using an ECL Advance Western Blotting Detection Kit (GE Healthcare). The band images were digitally captured with a FluorChem SP imaging system (Alpha Innotech, San Leandro, CA, USA) and the band intensities were quantified using a public domain software Image J (NIH Image, Bethesda, MD). The relative intensity of the individual proteins was expressed as the ratio of β-actin and the corresponding total protein.

### Immunohistochemistry

RBECs on Transwell inserts were fixed with 95% ethanol-5% acetic acid for 10 min at −20°C (occludin) or with ethanol for 1 min at room temperature (claudin-5 and ZO-1). Cells were blocked with Blocking One, and incubated with primary antibodies against ZO-1 (1∶100), claudin-5 (1∶100) and occludin (1∶100) overnight at 4°C (all purchased from Invitrogen). Cells were then incubated with FITC-labeled anti rabbit IgG for ZO-1 (1∶200) or Cy3-labeled anti mouse IgG for claudin-5 and occludin (Jackson ImmunoResearch) for 1 h at room temperature. All samples were imaged using a fluorescence microscope (BZ-8000; Keyence, Osaka, Japan).

### Statistical Analysis

Results are shown as means ± SEM. Statistical analysis was performed using Student’s *t* test to compare two groups. The statistical significance of differences between groups was assessed by one-way and two-way analyses of variance (ANOVA) for factorial comparisons and by Bonferroni or Tukey-Kramer’s test for multiple comparisons. Differences were considered significant when *P* values were less than 0.05, using GraphPad Prism 5.0 (GraphPad, San Diego, CA, USA).

## Results

### BBB Properties in the Neonatal and Adult *in vitro* BBB Models

**Figure 1:**
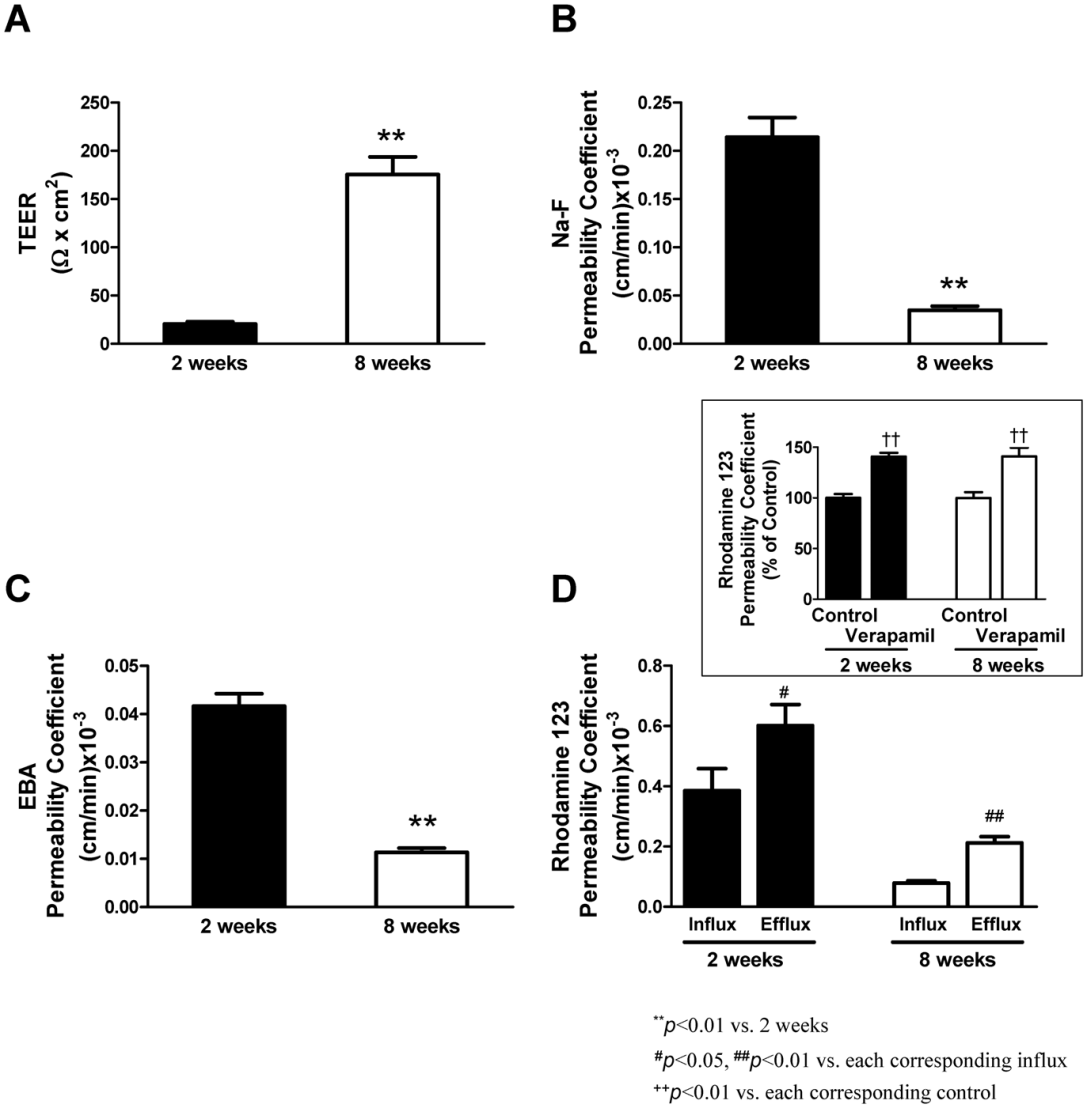
BBB properties in the neonatal and adult *in vitro* BBB models. The TEER (A), the permeability of Na-F (B) and EBA (C), and the influx (luminal-to-abluminal) and efflux (abluminal-to-luminal) of rhodamine 123 (D) in the neonatal (2 weeks, filled columns) and adult (8 weeks, open columns) BBB models. Inset in panel D shows the effect of verapamil on rhodamine 123 influx in both models. Data are means ± SEM (n = 11−12 from 3 separate experiments (A, B, and C), n = 12−19 from 3–5 separate experiments (D); n = 8−20 from 2–5 separate experiments (Fig. D inset)). ^**^ *P*<0.01, significantly different from the neonatal BBB model. ^#^ *P*<0.05, ^##^ *P*<0.01, significantly different from the influx of rhodamine 123.^ ++^ *P*<0.01, significantly different from each corresponding control.

Comparison between the neonatal (2 weeks, filled columns) and adult (8 weeks, open columns) *in vitro* BBB models for the barrier function and P-gp activity is shown in Fig. 1. TEER values increased, reached a peak during a period from 3 to 4 days after co-culture and decreased to the levels seen at the start of co-culture in the adult BBB model. This time course of TEER values was also observed in the neonatal BBB model even though peak TEER values were extremely low compared with those of the adult model. At a peak, TEERs of the neonatal and adult BBB models were 20.6±2.2 and 175.5±18.3 Ω×cm^2^, respectively (Fig. 1A). Permeability coefficients of the Na-F for the neonatal and adult BBB models were 0.21±0.02 and 0.035±0.004 (cm/min)×10^−3^, respectively (Fig. 1B). The EBA permeability coefficients for the neonatal and adult BBB models were 0.042±0.003 and 0.011±0.001 (cm/min)×10^−3^, respectively (Fig. 1C). The adult BBB model showed higher TEER values and lower permeability coefficients for Na-F and EBA than the neonatal BBB model. When the functional activities of P-gp were tested using rhodamine 123, the neonatal and adult BBB models, showed the abluminal to luminal (brain-to-blood) transport of rhodamine 123 was 1.6- and 2.7-fold higher, respectively, than blood-to-brain transport (Fig. 1D). When the BBB models were treated with verapamil (20 µM), the luminal to abluminal transport of rhodamine 123, in the neonatal and adult models, was significantly increased to 140.0±3.8 and 140.9±8.6% of control, respectively (Fig. 1D; inset). Therefore the brain-to-blood transport of rhodamine 123 in the adult BBB model was significantly higher than in the neonatal BBB model.

### Expression of the Tight Junction-associated Proteins and P-gp in the RBECs

**Figure 2:**
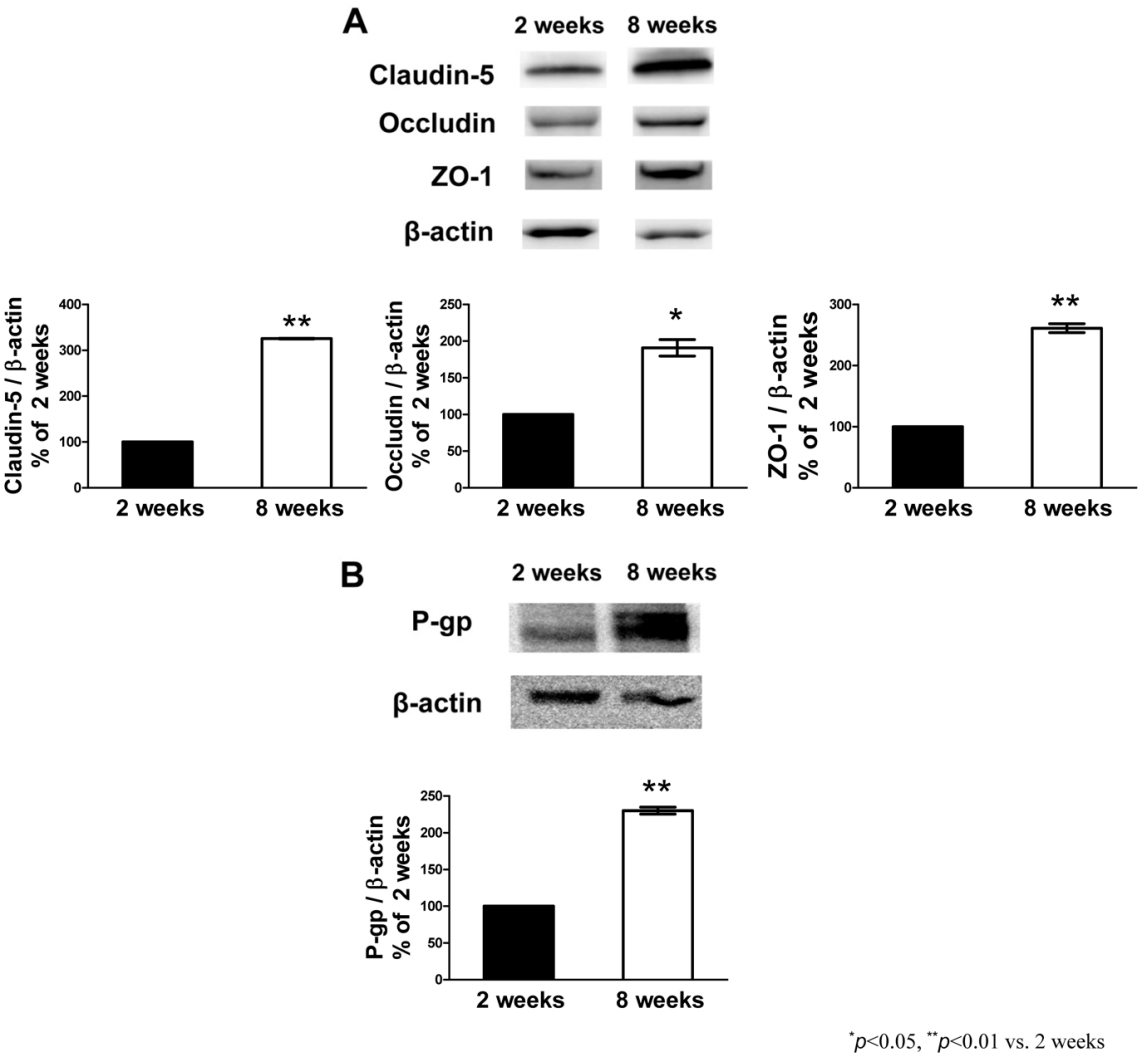
Expression of the tight junction-associated proteins and P-gp in the RBECs prepared from the neonatal and adult rats. Representative images of western blots showing the expression of claudin-5, occludin, ZO-1 (top panels in A), and P-gp (top panels in B) in RBECs prepared from the neonatal (2 weeks) and adult (8 weeks) rats. β-actin is used as a loading control. Band intensities were quantified by densitometry (bottom panels in A and B) and the data are expressed as a percentage of the neonatal (2 weeks) rats. Each bar indicates mean ± SEM (n = 3 from 3 separate experiments).^ *^ *P*<0.05, ^**^ *P*<0.01, significantly different from neonatal rats.

The levels of tight junction proteins (claudin-5, occludin, and ZO-1) and P-gp in the RBECs prepared from 2- and 8-week-old rats were determined by Western blot (Fig. 2). The levels of all these proteins in the adult RBECs were much higher than those in the neonatal RBECs.

### Distribution of Tight Junction-associated Proteins in RBECs from the *in vitro* BBB Models

**Figure 3:**
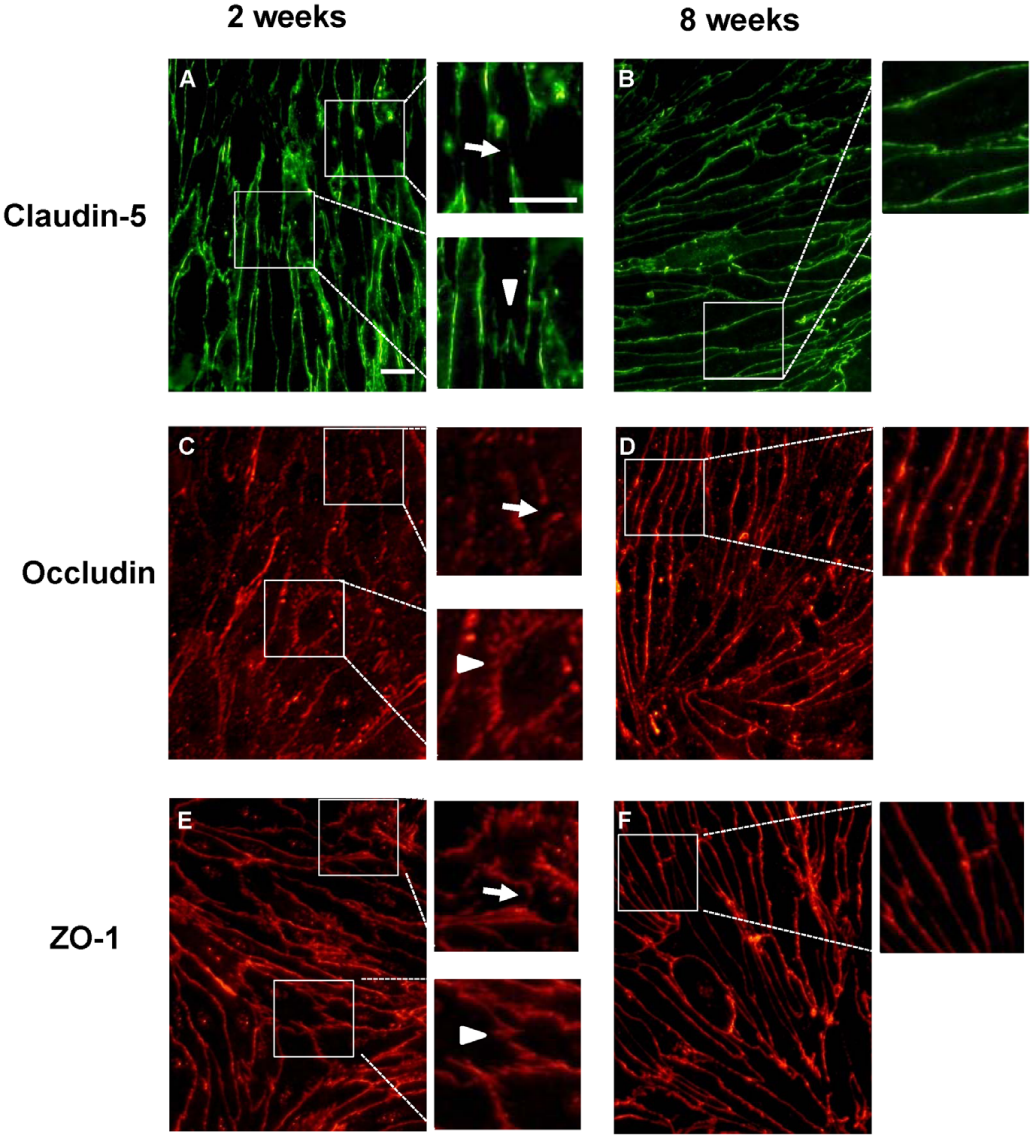
Distribution of tight junction-associated proteins in RBECs in neonatal and adult *in vitro* BBB models. Representative photographs showing immunofluorescent staining for claudin-5 (A & B), occludin (C & D) and ZO-1 (E & F) in RBECs on the inserts of neonatal (left panels) and adult (right panels) BBB models. Arrows indicate discontinuous lines and arrowheads indicate a zipper-like or zigzag shape distribution of tight junction-associated proteins. Inset panels show higher-magnification images of the squared region shown in each panel. Scale bars: 20 µm.

In RBECs from the adult BBB model, claudin-5, occludin and ZO-1 immunoreactivities were strongly expressed in the vicinity of cell borders, showing a linear shape along cell junctions (Fig. 3B, 3D and 3F; right panels). Conversely, immunostaining for the tight junction-associated proteins in RBECs from the neonatal BBB model, showed a discontinuous distribution of these proteins along cell borders. Compared with adult, neonatal RBECs showed a zipper-like or zigzag shape (Fig. 3; left panels).

### Comparison between *in vivo* and *in vitro* Drug Permeability and Age-related Variations in Drug Permeability

**Figure 4:**
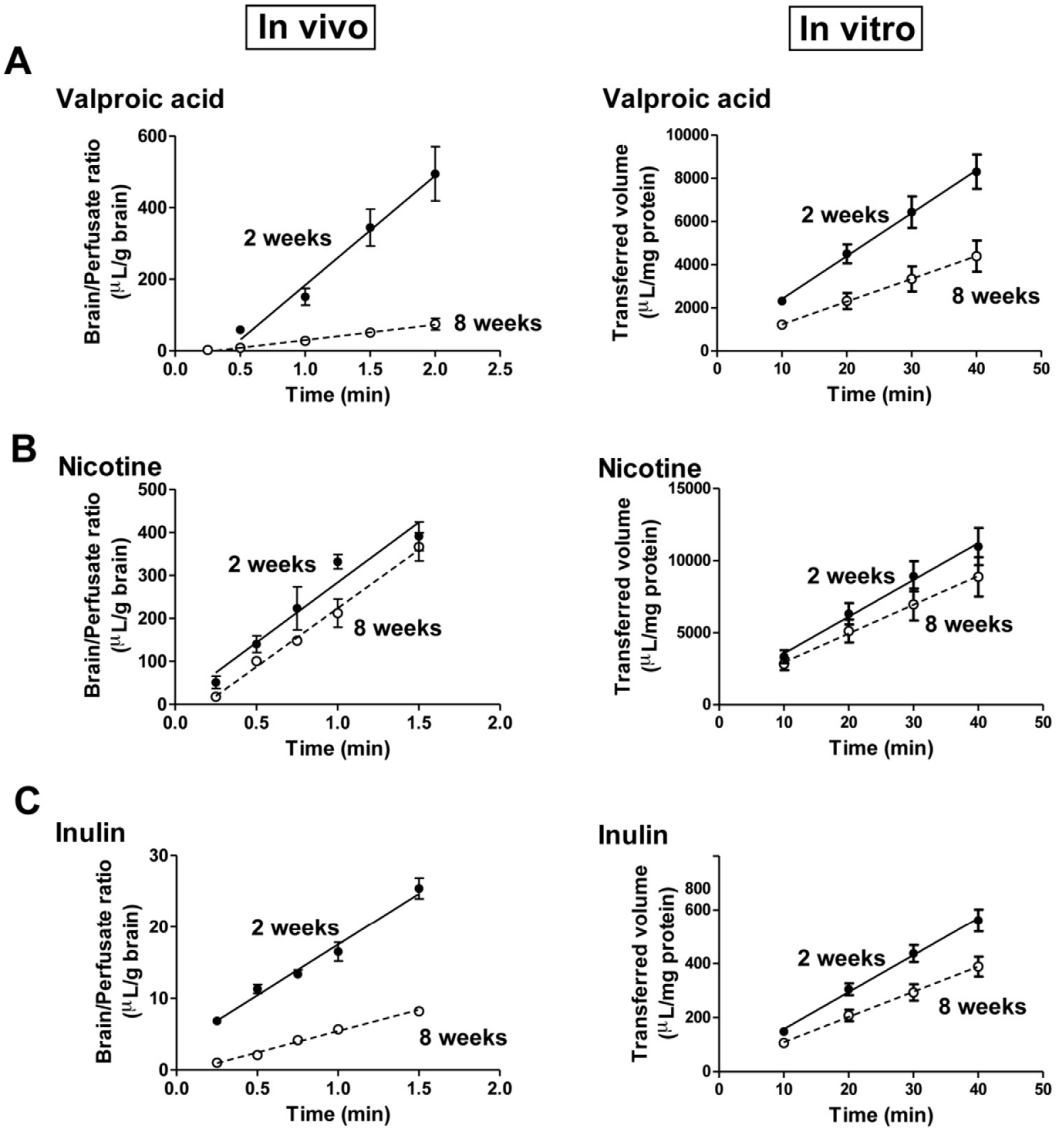
Time-courses of the BBB permeability to valproic acid, nicotine and inulin in the neonatal and adult rats (*in vivo*) and the neonatal and adult BBB models (*in vitro*). The time-courses of the brain/perfusate ratios of valproic acid, nicotine and inulin in the neonatal (2 weeks, filled circles) and adult (8 weeks, open circles) rats (*in vivo*) (left panels in A, B and C, respectively). The time-courses of the transferred volume of valproic acid, nicotine and inulin in the neonatal (2 weeks, filled circles) and adult (8 weeks, open circles) BBB models (*in vitro*) (right panels in A, B and C, respectively). Data are means ± SEM (n = 3−6 (*in vivo*) and n = 4−12 from 2–3 separate experiments (*in vitro*)).

Left panels of Fig. 4A, 4B and 4C show the time-courses for the ratios of tracer concentrations in the brain to those in the perfusate (apparent distribution volume) of valproic acid, nicotine and inulin, respectively, in the neonatal (2 weeks, filled circles) and adult (8 weeks, open circles) rats (*in vivo*). For these drugs, the distribution volumes increased linearly with time up to 120 s. The influx rate (Kin) was evaluated from the brain tracer uptake in the perfusion period of 120 s. Right panels of Fig. 4A, 4B and 4C show the time-courses for the transferred volume of valproic acid, nicotine and inulin, respectively, in the neonatal (2 weeks, filled circles) and adult (8 weeks, open circles) BBB models (*in vitro*). For these drugs, the transferred volumes increased linearly with time up to 40 min. The permeability clearance (PS_trans_) was evaluated from the transferred volume in the exposure periods of 10, 20, 30 and 40 min.

**Figure 5:**
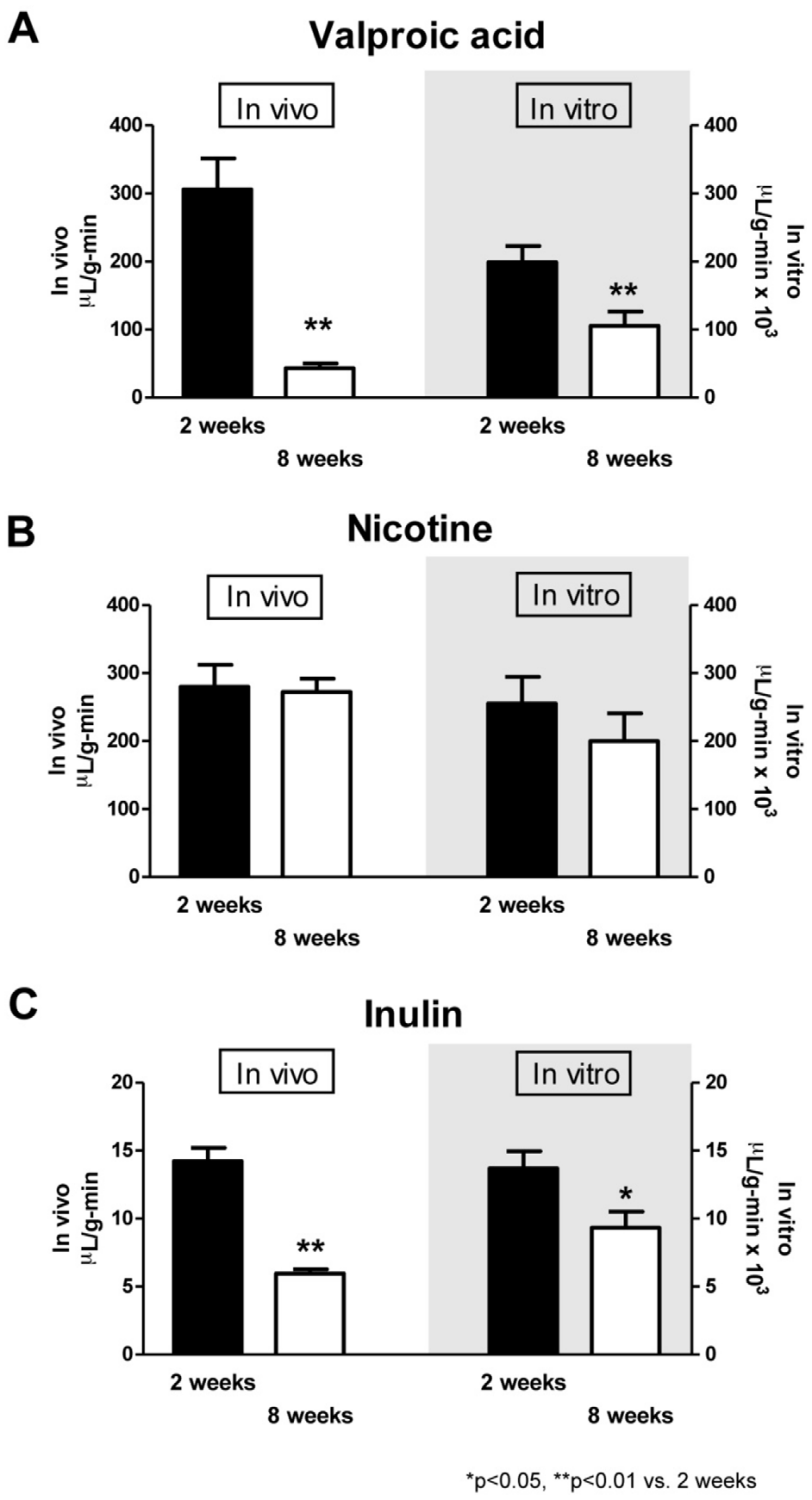
The influx rates of drugs in the neonatal and adult rats (*in vivo*) and the permeability clearance of drugs in the neonatal and adult BBB models (*in vitro*). The permeability of drugs (valproic acid (A), nicotine (B), inulin (C)) is expressed as the influx rate in the neonatal (2 weeks, filled columns) and adult (8 weeks, open columns) rats (*in vivo*: left panels) and the permeability clearance in the neonatal (2 weeks, filled columns) and adult (8 weeks, open columns) BBB models (*in vitro*: right panels). The influx rate (Kin) was evaluated from the brain tracer uptake in the perfusion period of 120 s and the permeability clearance (PS_trans_) was evaluated from the transferred volume in the exposure periods of 10, 20, 30 and 40 min. Data are means ± SEM (n = 3−6, *in vivo*; and n = 4−12 from 2–3 separate experiments, *in vitro*). ^*^ *P*<0.05, ** *P*<0.01, significantly different from neonatal rats (*in vivo*) or neonatal BBB model (*in vitro*).

As shown in Fig. 5, the *in vivo* Kin values of [^3^H] valproic acid, [^3^H] nicotine and [^14^C] inulin were 305.7±45.7, 279.8±32.2 and 14.2±1.0 µL/g·min, respectively, in the neonatal rats (filled columns), and 43.2±6.9, 272.2±19.8 and 6.0±0.31 µL/g·min, respectively, in the adult rats (open columns). In the neonatal rats, the *in vivo* influx rates (Kin) of [^3^H] nicotine and [^3^H] valproic acid showed 20- to 22-fold higher values than that of [^14^C] inulin. In the adult rats, the Kin of [^3^H] nicotine showed 6- and 45-fold higher values than Kin of [^3^H] valproic acid and Kin of [^14^C] inulin, respectively. The Kin values of [^3^H] nicotine and [^3^H] valproic acid in the neonatal rats were almost the same as that of [^3^H] nicotine in the adult rats. The neonatal rats showed 7- and 2-fold higher values than the adult rats in the Kin of [^3^H] valproic acid and that of [^14^C] inulin, respectively. In the *in vitro* BBB models, the PS_trans_ values of [^3^H] valproic acid, [^3^H] nicotine and [^14^C] inulin were 198.7±24.2×10^3^, 255.1±39.2×10^3^ and 13.7±1.3×10^3^ µL/g·min, respectively, in the neonatal BBB model, and 105.4±21.0×10^3^, 199.9±40.9×10^3^ and 9.3±1.2×10^3^ µL/g·min, respectively, in the adult BBB model. In both neonatal and adult BBB models, the highest PS_trans_ value was observed for the permeability clearance of [^3^H] nicotine; this value of the neonatal BBB model was an approximately that of the adult BBB model. In the neonatal BBB model, the PS_trans_ values of [^3^H] nicotine and [^3^H] valproic acid were 19- and 15-fold higher than that of [^14^C] inulin, respectively. In the adult BBB model, the PS_trans_ values of [^3^H] nicotine and [^3^H] valproic acid were 22- and 11-fold higher than that of [^14^C] inulin, respectively. The PS_trans_ values of [^3^H] valproic acid and [^14^C] inulin in the neonatal BBB model were 2- and 1.5-fold of each corresponding value in the adult BBB model.

*In vitro* and *in vivo*, the permeability of [^3^H] valproic acid and [^14^C] inulin markedly decreased with age. The [^3^H] nicotine permeability was very high *in vitro* and *in vivo*; this high permeability was not influenced by age. Age-related variations in the other drugs’ permeability were observed both *in vivo* and *in vitro*.

## Discussion

In this study, we first attempted to establish neonatal and adult *in vitro* BBB models using BMECs isolated from neonatal and adult rats. The time course of the TEERs suggests that the analytical window of our BBB models may be a period of 2 days from 3 to 4 days after co-culture. Various species have been used to reconstitute the *in vitro* BBB model. The advantage of the rodent BBB model is that it enables comparison with *in vivo* permeability data. Although both neonatal and adult BBB models consisted of triple, co-culturing of BMECs, pericytes and astrocytes, only BMECs were prepared from different aged rats. The pericytes and astrocytes used in both BBB models were isolated from 3-week-old and 1- to 3-day-old rats, respectively. At present, experimental methods have not yet been established to prepare primary cultures of pericytes or astrocytes isolated from neonatal and adult brains.

Previous work demonstrated that BMECs isolated from 10-day-old or 5-week-old mice showed age-related differences in the expression of monocalboxylate transporter 1 and glucose transporter 1 [32]. This work strongly suggested the possibility of reproducing the age-related BBB functional differences *in vitro*. We found that the neonatal BBB model showed higher permeability for Na-F and EBA and lower TEER compared with the adult BBB model (Fig. 1A, 1B and 1C). Moreover, in the neonatal and adult BBB models, the efflux rate of rhodamine 123 was 1.6- and 2.7-fold higher, respectively, than the influx rate (Fig. 1D). In the presence of the P-gp inhibitor, verapamil, the influx rate of rhodamine 123 was significantly increased by 40% of control in both the neonatal and adult BBB models (Fig. 1D; inset). These results suggest that P-gp expressed in the neonatal BBB model is likely functional, although its activity is low compared with the adult BBB model. These age-related differences in the transendothelial barrier and P-gp functions between the neonatal and adult BBB models were consistent with the expression levels of tight junction proteins and P-gp (Fig. 2), and varied morphological distribution of tight junction proteins along cell borders (Fig. 3), in BMECs obtained from neonatal and adult rats. These data indicated that the age-related functional differences in the permeability of BMECs were retained even if they were cultured and grown in the same *in vitro* condition. Using a monoculture system, we confirmed that the neonatal BMECs showed lower TEER (Ω×cm^2^) and higher permeability for Na-F and EBA (permeability coefficient, (cm/min) ×10^–3^) than the adult BMECs (TEER: 9.37±1.0 (neonatal), 77.7±8.1 (adult), Na-F: 0.40±0.04 (neonatal), 0.042±0.001 (adult), EBA: 0.056±0.003 (neonatal), 0.011±0.002 (adult), n = 4−12 each). Therefore, the essential properties of the BBB in the neonatal BMECs are different from those of the adult BMECs *in vitro*, although the culture media supplements (hydrocortisone, insulin-transferrin-sodium selenite media supplement and plasma-derived serum) could influence the induction and maintenance of the endothelial barrier properties during the period when BMECs were growing on the transwell inserts. The possibility of age-related differences in the sensitivity to the inducible factors influencing the BBB properties has to be considered.

Next, we determined whether the BBB permeability to drugs depends on age, and then we evaluated the usefulness of our neonatal BBB model to predict the *in vivo* BBB permeability. We measured the *in vitro* and *in vivo* BBB permeability to valproic acid, nicotine and inulin. The reasons for using the brain perfusion technique for the *in vivo* BBB model are (1) to equalize the concentrations of the test compounds in the fluid of the brain artery between neonatal and adult rats and (2) to prevent serum protein from binding to the test compounds. The three compounds included BBB impermeable (inulin) and BBB-permeable (nicotine and valproic acid) compounds, that are known to be distributed by passive diffusion. Valproic acid has also been reported to be transported by P-gp, an active efflux transporter [33], [34]. As shown in Fig. 4, the time-courses of the brain/perfusate ratios of the test compounds in the neonatal and adult rats could be classified into 2 patterns. For valproic acid and inulin, the intersections of the regression lines indicated that differences between the neonatal and adult rats in the brain/perfusate ratios gradually increased with time. For nicotine, the parallel regression lines indicated that the differences in the brain/perfusate ratios between the neonatal and adult rats did not change with time. These findings suggest that the BBB permeability to drugs apparently depends on age. The lower expression levels of tight junction proteins and P-gp in the neonatal BBB could explain these differences between valproic acid and inulin. No difference was observed in the BBB permeability for nicotine between the neonatal and adult rats. Nicotine, a lipophilic molecule, is capable of penetrating the BBB through passive diffusion through the endothelial cellular membrane. Similar results were observed in the transferred volume-time profile of these compounds in the neonatal and adult BBB models. Fig. 5 shows a comparison of the *in vivo* influx rate with the *in vitro* PS_trans_ value. The latter values (PS_trans_) of the *in vitro* BBB models were normalized with the protein content of the BMECs in each well expressed as µL/g-min. Although, overall, the PS_trans_ values *in vitro* were 1000 times higher than the Kin values *in vivo*, the PS_trans_ values obtained from the neonatal or adult BBB models were well correlated with the influx rate obtained from the neonatal or adult rats. These results suggest that our *in vitro* neonatal and adult BBB models can predict the age-dependent BBB properties and BBB permeability when testing compounds *in vivo*.

In conclusion, we established a neonatal BBB model using BMECs isolated from neonatal (2 weeks old) rats. We showed that the age-related barrier properties of BMECs were retained *in vitro*. With reference to the drug permeability, a good relationship was observed between the influx rate obtained from the neonatal rats and the PS values obtained from the *in vitro* neonatal BBB model. Therefore, this neonatal BBB model could be a good tool for high throughput screening to predict age-related BBB permeability *in vivo* of compounds including therapeutic drugs, drug candidates and environmental toxins.
